# FGF23 protects osteoblasts from dexamethasone-induced oxidative injury

**DOI:** 10.18632/aging.103689

**Published:** 2020-10-14

**Authors:** Feng Ji, Xiaohui Hu, Wenhao Hu, Yue-Dong Hao

**Affiliations:** 1 Department of Orthopedics, The Affiliated Huaian No.1 People’s Hospital of Nanjing Medical University, Huaian, China

**Keywords:** FGF23, FGFR1, osteoblasts, dexamethasone, Akt

## Abstract

Dexamethasone (DEX) can exert a cytotoxic effect on cultured osteoblasts. The current study explored the potential osteoblast cytoprotective effect of fibroblast growth factor 23 (FGF23). In OB-6 human osteoblastic cells and primary murine osteoblasts, FGF23 induced phosphorylation of the receptor FGFR1 and activated the downstream Akt-S6K1 signaling. FGF23-induced FGFR1-Akt-S6K phosphorylation was largely inhibited by FGFR1 shRNA, but augmented with ectopic FGFR1 expression in OB-6 cells. FGF23 attenuated DEX-induced death and apoptosis in OB-6 cells and murine osteoblasts. Its cytoprotective effects were abolished by FGFR1 shRNA, Akt inhibition or Akt1 knockout. Conversely, forced activation of Akt inhibited DEX-induced cytotoxicity in OB-6 cells. Furthermore, FGF23 activated Akt downstream nuclear-factor-E2-related factor 2 (Nrf2) signaling to alleviate DEX-induced oxidative injury. On the contrary, Nrf2 shRNA or knockout almost reversed FGF23-induced osteoblast cytoprotection against DEX. Collectively, FGF23 activates FGFR1-Akt and Nrf2 signaling cascades to protect osteoblasts from DEX-induced oxidative injury and cell death.

## INTRODUCTION

Dexamethasone (DEX) is a medication routinely prescribed to patients with chronic inflammatory and auto-immune diseases [1]. In United States an estimated 30 million patients are currently taking DEX [2]. However studies have shown that long-term and/or excessive DEX consumption could lead to osteoporosis (or even osteonecrosis) [3–5]. Over 30% or more DEX-taking patients will suffer bone fractures [3], presenting with decreased number of osteoblasts and increased osteoblast cell apoptosis in the bones [3–5]. DEX can exert a cytotoxic effect to cultured osteoblasts [6–8]. Our group has been dedicated to exploring the pathological mechanisms of DEX-induced injury to osteoblasts/osteoblastic cells [9–15].

Fibroblast growth factor 23 (FGF23), a phosphaturic factor, is a member of the endocrine FGF19 family. It is produced by osteoblasts in the bone [16–18]. Its known function is to promote renal phosphate wasting by suppressing expression of renal tubular sodium phosphate cotransporter type IIa (NPT2a) [16, 19]. FGF23, like other FGFs, signals through the FGF receptors (FGFRs), mainly FGFR1 [20]. FGF23-FGFR binding affinity is high [16, 19, 20], which will induce FGFR1 phosphorylation, thus recruiting multiple adaptor proteins to activate PI3K-Akt and other downstream cascades [16, 19, 20]. FGF23 can be produced by osteoblasts [21]. Studies have suggested autocrine and paracrine actions of FGF23 in osteoblasts [22]. FGF23 has been shown to promote human cancer cell survival and progression [23]. Whether FGF23 can activate FGFR1 in osteoblasts and offer cytoprotection against DEX-induced cytotoxicity have not been studied thus far.

The nuclear-factor-E2-related factor 2 (Nrf2) cascade is recognized as one of the most important endogenous antioxidant mechanism [24–27]. Without stimulation (basal conditions), Nrf2 association with its suppressor protein and also a stress sensor Keap1, subjected to ubiquitination and proteasomal degradation by Cullin 3 E3 ubiquitin ligase [24–27]. Nrf2 activation will lead to Keap1-Nrf2 disassociation, Nrf2 protein cytosol accumulation and following nuclear translocation. It will then lead to transcription and expression of the antioxidant response element (ARE) genes, including phase 2 detoxifying enzymes and antioxidant proteins [24–27]. Expression of these genes, including *heme oxygenase-1 (HO-1)*, *NAD(P)H:quinone oxidoreductase 1 (NQO1)*, *γ-glutamyl cysteine ligase catalytic subunit* (GCLC)*,* and *the modifier subunit* (*GCLM*) [28], will provoke significant antioxidant and cytoprotective activity [29, 30]. We will show here that FGF23 activates Nrf2 signaling, lying downstream of FGFR1-Akt, thereby protecting osteoblasts from DEX-induced oxidative injury and cell death.

## RESULTS

### FGF23 activates FGFR1-Akt-S6K1 and Erk1/2 signalings in osteoblasts

**Figure 1:**
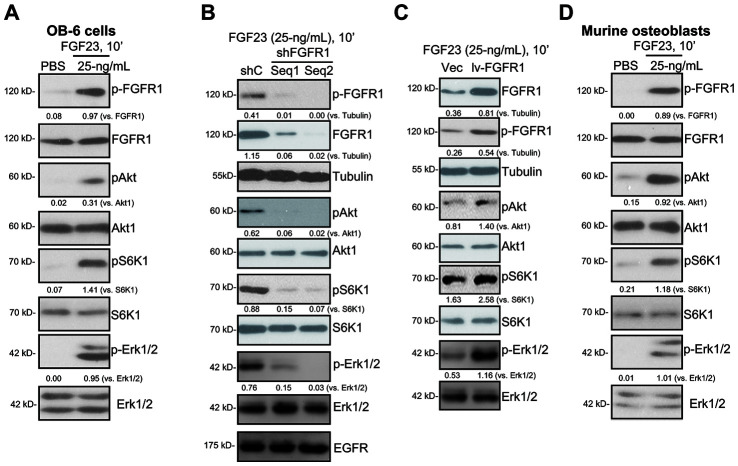
**FGF23 activates FGFR1-Akt-S6K1 and Erk1/2 signalings in osteoblasts.** The differentiated OB-6 human osteoblastic cells (**A**) or the primary murine osteoblasts (**D**) were treated with FGF23 (25 ng/mL) or PBS (the vehicle control) for 10 min, total cell lysates were collected, with the expression of listed proteins tested by Western blotting (**A** and **D**). Stable OB-6 cells, with the applied FGFR1 shRNA (“shFGFR1-Seq1/2”, two non-overlapping sequences), the scramble control shRNA (“shC”), the lentiviral FGFR1-expressing construct (“lv-FGFR1”), or the empty vector (“Vec”), were treated with FGF23 (25 ng/mL) for 10 min, expression of listed proteins in total cell lysates were tested (**B** and **C**). For Western blotting assays, the same set of lysate samples were run in sister gels to test different proteins (same for all Figures). The exact same amount of protein lysates, 40 μg lysates per lane, were loaded in each lane (same for all Figures). The listed proteins were quantified and normalized to the loading controls (same for all Figures). Each experiment was repeated three times and similar results were obtained.

In the differentiated OB-6 human osteoblastic cells, FGFR1, the main functional receptor of FGF23 (Figure 1A), is expressed. Significantly, FGF23 (25 ng/mL) robustly induced FGFR1 phosphorylation in OB-6 cells (Figure 1A). Furthermore, phosphorylation and activation of FGFR1’s downstream cascades, Akt and S6K1, was detected in FGF23-stimulated OB-6 cells (Figure 1A). FGF23 induced Erk1/2 phosphorylation in OB-6 osteoblasts as well (Figure 1A).

To silence FGFR1, two lentiviral FGFR1 shRNAs, against non-overlapping sequences of FGFR1 (“shFGFR1-Seq1/Seq2”), were individually transduced to OB-6 cells. Via selection by puromycin-containing medium, the stable cells were established. Testing FGFR1 expression, we show that the applied FGFR1 shRNAs induced over 90% knockdown of FGFR1 in stable cells (Figure 1B). Importantly, FGF23-induced phosphorylation of FGFR1, Akt, S6K1 and Erk1/2 was largely inhibited by the applied FGFR1 shRNAs (Figure 1B). The applied FGFR1 shRNAs did not alter expression of EGFR (Figure 1B). On the contrary, a lentiviral FGFR1-expressing construct (“lv-FGFR1”) was transduced to OB-6 cells, leading to FGFR1 overexpression (Figure 1C). Ectopic overexpression of FGFR1 augmented FGF23-induced phosphorylation of FGFR1, Akt, S6K1 and Erk1/2 in OB-6 cells. These results further supported the functional FGFR1 expression in OB-6 cells.

In the primary murine osteoblasts, FGFR1 expression was also detected (Figure 1D). Further, FGF23 treatment induced phosphorylation of FGFR1, Akt, S6K1 and Erk1/2 in the murine osteoblasts (Figure 1D). Together, these results show that FGF23 activated FGFR1-Akt-S6K1 and Erk1/2 signalings in the osteoblasts.

### FGF23 protects osteoblasts from DEX-induced cell death and apoptosis

**Figure 2:**
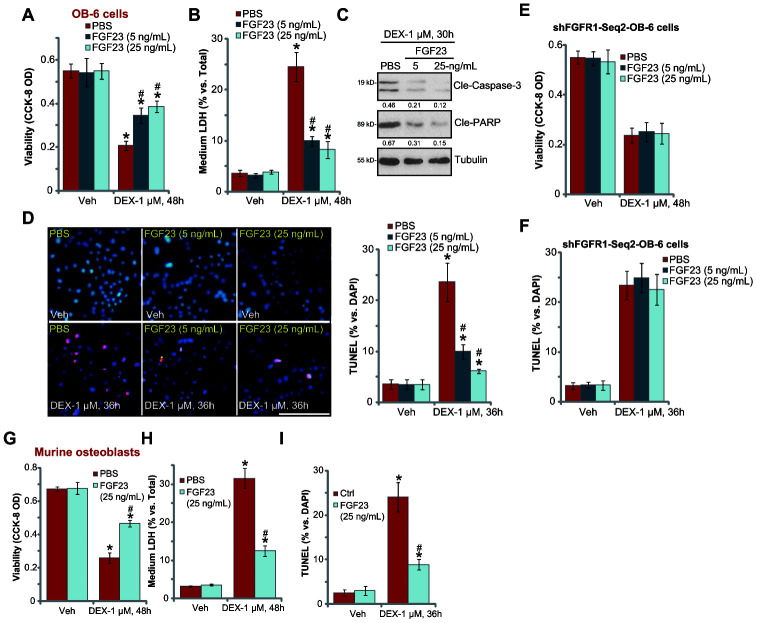
**FGF23 protects osteoblasts from DEX-induced cell death and apoptosis.** The differentiated OB-6 human osteoblastic cells (**A**–**D**) or the primary murine osteoblasts (**G**–**I**) were pretreated with applied concentration of FGF23 (5 or 25 ng/mL) or vehicle control (PBS) for 2h, followed by dexamethasone (DEX, 1 μM) stimulation, cells were further cultured for the indicated time periods, cell viability and cell death were tested by CCK-8 (**A** and **G**) or medium LDH release (**B** and **H**) assays respectively; expression of the listed apoptosis-associated proteins was shown (**C**), with cell apoptosis tested by nuclear TUNEL staining (**D** and **I**). Stable OB-6 cells with the applied FGFR1 shRNA (“shFGFR1-Seq2”) were pretreated with FGF23 (5 or 25 ng/mL) or PBS for 2h, followed by DEX (1 μM) stimulation, cells were further cultured for 48h, with cell viability (**E**) and apoptosis (**F**) tested similarly. Data were mean ± standard deviation (SD, n=5). “Veh” stands for vehicle control for DEX. * p<0.05 *vs.* “Veh” cells with PBS pretreatment. ^#^ p<0.05 *vs.* DEX-treated cells with PBS pretreatment. Each experiment was repeated three times and similar results were obtained. Bar=100 μm (**D**).

Next, experiments were performed to test the potential effect of FGF23 in DEX-treated osteoblasts. OB-6 cells were treated with DEX. In line with the previous findings [9, 31–33], DEX treatment (1 μM [31, 32]) induced significant cell viability (CCK-8 OD) reduction (Figure 2A) and cell death (medium LDH release, Figure 2B) in OB-6 cells, which were largely attenuated by pretreatment of FGF23 (at 5-25 ng/mL, 2h pretreatment) (Figure 2A and 2B). In OB-6 cells, DEX treatment induced apoptosis activation, which was evidenced by cleavages of caspase-3-PARP (poly (ADP-ribose) polymerase) (Figure 2C) and increased nuclear TUNEL staining (Figure 2D). The pro-apoptotic activity of DEX was significantly inhibited with FGF23 pretreatment (Figure 2C and 2D). In FGFR1-silenced OB-6 cells (by shFGFR1-Seq2, see Figure 1), FGF23 pretreatment was completely ineffective against DEX-induced cell death (Figure 2E) and apoptosis (Figure 2F). Therefore, FGFR1 activation is required for FGF23-induced osteoblast cytoprotection.

In the primary murine osteoblasts DEX treatment similarly induced viability reduction (Figure 2G), cell death (Figure 2H), and apoptosis activation (Figure 2I). FGF23 (25 ng/mL, 2h pretreatment) similarly attenuated DEX-induced cytotoxicity in murine osteoblasts (Figure 2G–2I). These results demonstrated that FGF23 pretreatment potently inhibited DEX-induced cytotoxicity in OB-6 cells and primary murine osteoblasts. FGF23 single treatment did not alter viability and apoptosis in the tested osteoblasts.

### Akt activation mediates FGF23-induced osteoblast cytoprotection against DEX

**Figure 3:**
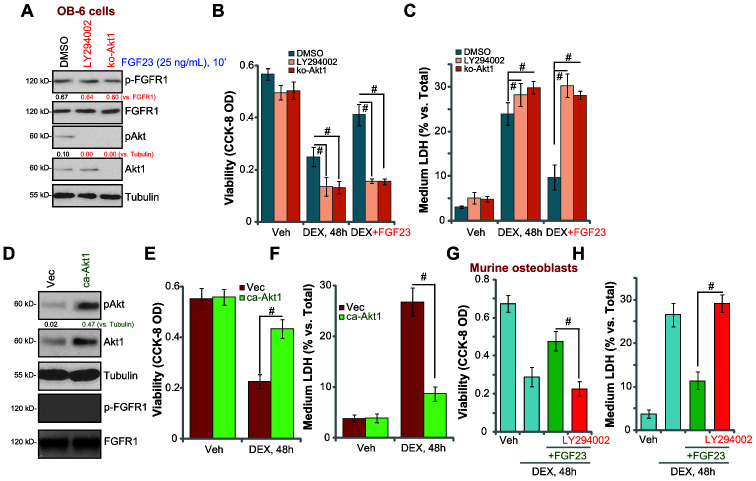
**Akt activation mediates FGF23-induced osteoblast cytoprotection against DEX.** The control OB-6 osteoblastic cells, pre-treated with LY294002 (500 nM, 30 min pretreatment) or the vehicle control (0.1% DMSO), as well as the stable OB-6 cells with the CRISPR/Cas9-Akt1-KO construct (“ko-Akt1”), were treated with FGF23 (25 ng/mL), after 10 min expression of the listed proteins in total cell lysates were shown (**A**). Alternatively two hours after the FGF23 treatment, cells were treated with dexamethasone (DEX, 1 μM) or the vehicle control (“Veh”), cell viability and cell death were tested by CCK-8 (**B**) or medium LDH release (**C**) assays, respectively. The stable OB-6 cells with the constitutively-active Akt1 construct (caAkt1) or the empty vector (“Vec”) were subjected to Western blotting assays to test listed proteins (**D**). Cells were treated with DEX (1 μM) or the vehicle control (“Veh”), after 48h cell viability (**E**) and cell death (**F**) were tested. The primary murine osteoblasts were pretreated with LY294002 (500 nM, 30 min pretreatment), followed by FGF23 (25 ng/mL) treatment for 2h, cells were further stimulated with DEX (1 μM) for 48h, cell viability (**G**) and death (**H**) were tested. Data were mean ± standard deviation (SD, n=5). ^#^ p<0.05. Each experiment was repeated three times and similar results were obtained.

Akt is a key pro-survival factor, protecting cells against various stress stimulations [34]. To block Akt activation, the Akt inhibitor LY294002 was applied. Furthermore, the CRISPR/Cas9 gene-editing method was utilized to completely knockout (KO) Akt1 [35] in OB-6 cells (Figure 3A). As shown, LY294002 or Akt1-KO almost blocked FGF23-induced Akt phosphorylation in OB-6 cells, while leaving upstream FGFR1 expression/phosphorylation unchanged (Figure 3A). Importantly, with LY294002 or Akt1-KO, FGF23 (25 ng/mL) was unable to protect OB-6 cells from DEX-induced viability reduction (Figure 3B) and cell death (Figure 3C). Therefore, Akt activation is required for FGF23’s osteoblast cytoprotective actions. LY294002 or Akt1 knockout alone intensified DEX-induced cytotoxicity in OB-6 cells (Figure 3B and 3C), suggesting that the basal Akt activation is important for the survival of DEX-treated OB-6 cells.

Next a constitutively-active Akt1 (caAkt1, from Dr. Zhang [36]) was transduced to OB-6 cells, increasing Akt activation without FGF23 stimulation (Figure 3D). The caAkt1, as expected, did not induce FGFR1 phosphorylation (Figure 3D). In OB-6 cells the caAkt1 largely alleviated DEX-induced viability reduction (Figure 3E) and cell death (Figure 3F). Therefore, forced activation of Akt attenuated DEX-induced cytotoxicity in OB-6 cells, mimicking FGF23-induced actions. In the primary murine osteoblasts, the Akt inhibitor LY294002 almost abolished FGF23-induced anti-DEX actions (Figure 3G and 3H). Taken together, these results imply that Akt activation mediated FGF23-induced osteoblast cytoprotection against DEX. Contrarily, Erk inhibitors, PD98059 and U0126, had no significant effect on FGF23-induced osteoblast cytoprotection against DEX (Supplementary Figure 1A and 1B).

### FGF23 inhibits DEX-induced oxidative stress in osteoblasts

**Figure 4:**
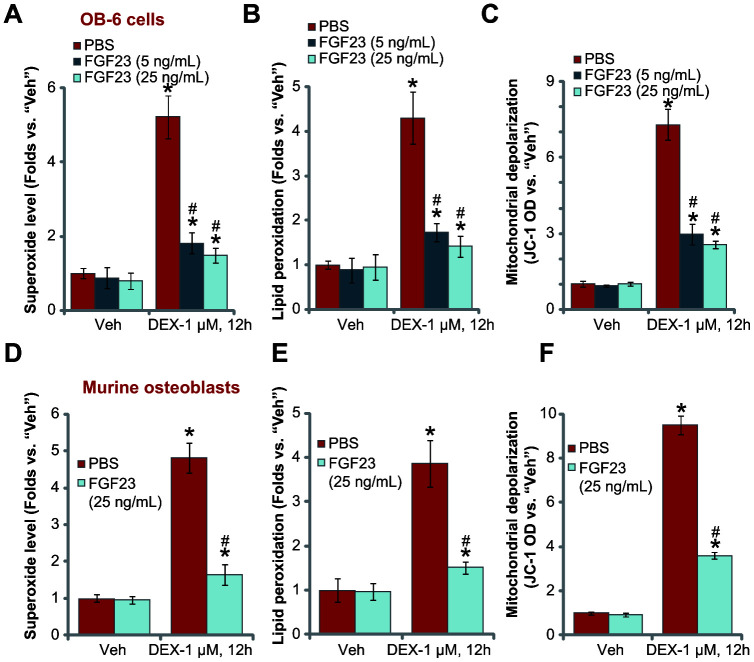
**FGF23 inhibits DEX-induced oxidative stress in osteoblasts.** OB-6 osteoblastic cells (**A**–**C**) or the primary murine osteoblasts (**D**–**F**) were pretreated with applied concentration of FGF23 (5 or 25 ng/mL) or vehicle control (PBS) for 2h, followed by dexamethasone (DEX, 1 μM) stimulation, cells were further cultured for additional 12h, superoxide contents (**A** and **D**), lipid peroxidation intensity (**B** and **E**) and mitochondrial depolarization (JC-1 green fluorescence accumulation, **C** and **F**) were tested, with results normalized. Data were mean ± standard deviation (SD, n=5). “Veh” stands for vehicle control for DEX. * p<0.05 *vs.* “Veh” cells with PBS pretreatment. ^#^ p<0.05 *vs.* DEX-treated cells with PBS pretreatment. Each experiment was repeated three times and similar results were obtained.

Studies have shown that DEX induces reactive oxygen species (ROS) production and oxidative injury in osteoblasts, contributing to subsequent cell death and apoptosis [9, 10, 37–39]. Contrarily, inhibition of oxidative injury will efficiently protect osteoblasts from DEX [9, 10, 37–39]. The results above have shown that FGF23 alleviated DEX-induced cytotoxicity in osteoblasts, we thus tested its potential effect on DEX-induced oxidative injury. In OB-6 cells DEX treatment (1 μM, 12h) increased superoxide level (Figure 4A), which was accompanied by lipid peroxidation (Figure 4B) and mitochondrial depolarization (JC-1 green fluorescence accumulation, Figure 4C). Importantly, FGF23 pretreatment largely attenuated DEX-induced oxidative injury in OB-6 cells (Figure 4A–4C).

In the primary murine osteoblasts, FGF23 exerted a similar antioxidant activity, inhibiting DEX-induced superoxide accumulation (Figure 4D), lipid peroxidation (Figure 4E) and mitochondrial depolarization (Figure 4F). FGF23 treatment alone did not alter oxidative levels in the osteoblasts (Figure 4A–4F). These results clearly show that FGF23 inhibited DEX-induced oxidative stress in osteoblasts.

### FGF23 activates Nrf2 signaling in osteoblasts

**Figure 5:**
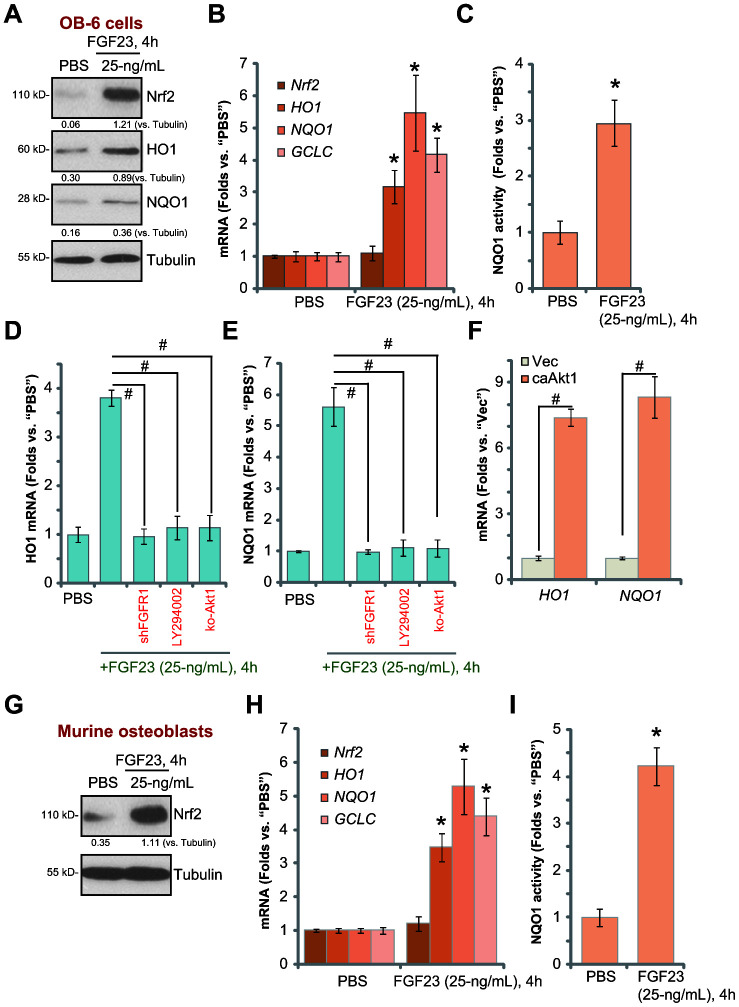
**FGF23 activates Nrf2 signaling in osteoblasts.** OB-6 osteoblastic cells (**A**–**C**) or the primary murine osteoblasts (**G**–**I**) were treated with FGF23 (25 ng/mL) for 4h, expression of listed genes in total cell lysates was tested by Western blotting and qPCR assays (**A**, **B**, **G** and **H**), with relative NQO1 activity tested as well (**C** and **I**). The control OB-6 cells, pre-treated with LY294002 (500 nM, 30 min pretreatment), as well as the stable OB-6 cells with the lentiviral FGFR1 shRNA (“shFGFR1”) or the CRISPR/Cas9-Akt1-KO construct (“ko-Akt1”), were treated with FGF23 (25 ng/mL) for 4h, expression of *HO1* and *NQO1* mRNA was shown (**D** and **E**). The relative *HO1* and *NQO1* mRNA expression in stable OB-6 cells with the constitutively-active Akt1 construct (caAkt1) or the empty vector (“Vec”) was tested (**F**). Data are presented as the mean ± standard deviation (n=5).* p<0.05 *vs.* PBS treatment. ^#^ p<0.05. Each experiment was repeated three times and similar results were obtained.

Studies have shown that forced activation of Akt in osteoblasts could induce Nrf2 cascade activation, inhibiting DEX-induced oxidative injury [10, 39]. Thus, we tested the potential effect of FGF23 on Nrf2 signaling. Western blotting assay results, Figure 5A, demonstrated that Nrf2 protein levels were significantly elevated in FGF23-treated OB-6 cells. The mRNA expression of Nrf2-dependent genes, including *HO1*, *NQO1* and *GCLC*, was significantly increased following FGF23 treatment in OB-6 cells, with *Nrf2 mRNA* levels unchanged (Figure 5B). HO1 and NQO1 protein levels were increased as well (Figure 5A). Additionally, the NQO1 activity was enhanced in FGF23-treated in OB-6 cells (Figure 5C). These results indicate that FGF23 activated Nrf2 signaling cascade, leading to stabilization of Nrf2 protein, expression of Nrf2-pathway genes and an increase of NQO1 activity in OB-6 cells.

Importantly, FGF23-induced mRNA expression of *HO1* and *NQO1* was almost completely blocked by FGFR1 shRNA (Seq-2, Figure 5D and 5E). LY294002 or Akt1 knockout (see Figure 3) abolished *HO1*-*NQO1* mRNA expression in FGF23-treated OB-6 cells (Figure 5D and 5E). On the contrary, *HO1* and *NQO1* mRNA levels were significantly increased in ca-Akt1-expressed OB-6 cells (Figure 5F). These results indicate that FGFR1-Akt activation is required FGF23-induced Nrf2 cascade activation.

In the primary murine osteoblasts FGF23 treatment induced Nrf2 protein stabilization (Figure 5G), increased mRNA expression of Nrf2-dependent genes (*HO1*, *NQO1* and *GCLC*) (Figure 5G and 5H), as well as NQO1 activity (Figure 5I). Taken together, these results show that FGF23 activated Nrf2 signaling in osteoblasts.

### Nrf2 silencing or KO abolishes FGF23-induced osteoblast cytoprotection against DEX

**Figure 6:**
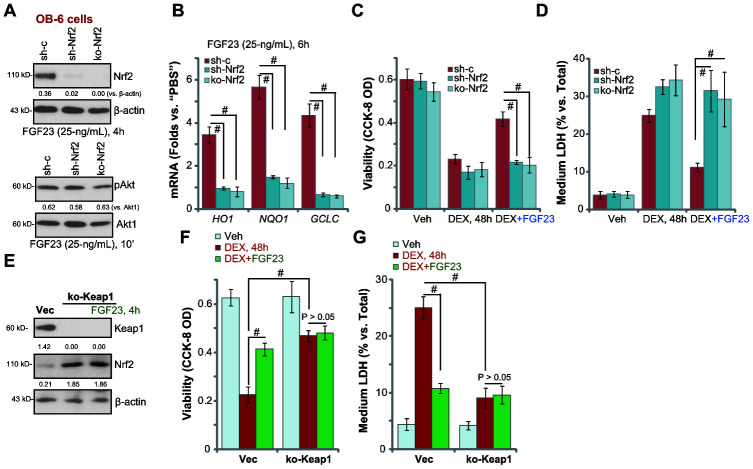
**Nrf2 silencing or KO abolishes FGF23-induced osteoblast cytoprotection against DEX.** Genetically-modified stable OB-6 cells with the lentiviral Nrf2 shRNA (“shNrf2) or the lentiCRISPR-GFP-Nrf2 KO construct (“ko-Nrf2”), as well as the parental control cells (“Ctrl”) were treated with FGF23 (25 ng/mL) for applied time periods, expression of listed mRNAs and proteins was shown (**A** and **B**); Cells were pretreated for 2h with FGF23 (25 ng/mL), followed by dexamethasone (DEX, 1 μM) stimulation for 48h, cell viability and death were tested by CCK-8 (**C**) and medium LDH release (**D**) assays, respectively. Stable OB-6 cells with CRISPR/Cas9-Keap1-KO construct (“ko-Keap1”) were treated with or without FGF23 (25 ng/mL) for 4h, control cells were tranduced with empty vector (“Vec”), expression of listed proteins was shown (**E**). Alternatively, cells were pretreated for 2h with FGF23 (25 ng/mL), followed by dexamethasone (DEX, 1 μM) stimulation for 48h, cell viability (**F**) and death (**G**) were tested. Data are presented as the mean ± standard deviation (n=5). ^#^ p<0.05. The experiments in this figure were repeated three times, and similar results were obtained.

To test whether Nrf2 cascade is important for FGF23-induced osteoblast cytoprotection against DEX, the Nrf2 shRNA lentiviral particles (from Dr. Xu [40]) were utilized, causing potent Nrf2 knockdown in FGF23-treated OB-6 cells (Figure 6A). Furthermore, a CRISPR-Cas9 Nrf2-KO construct (from Dr. Xu [40]) was utilized to completely knockout Nrf2 (Figure 6A). Nrf2 shRNA or KO did not alter FGF23-induced Akt activation (Figure 6A). FGF23-induced mRNA expression of Nrf2-dependent genes, *HO1*, *NQO1* and *GCLC*, was blocked by Nrf2 shRNA or KO (Figure 6B). Significantly, in Nrf2-silenced or Nrf2-KO OB-6 cells, FGF23 was unable to inhibit DEX-induced viability reduction (Figure 6C) and cell death (Figure 6D). Therefore, FGF23-induced osteoblast cytoprotection against DEX was abolished with Nrf2 silencing or KO.

Keap1 depletion should induce Nrf2 cascade activation and mimic FGF23-induced osteoblastic cytoprotection. Therefore, a lentiviral CRISPR/Cas9-Keap1-KO construct was transduced to OB-6 cells, and stable cells established with puromycin selection (“ko-Keap1” cells). CRISPR/Cas9-induced Keap1 depletion (Figure 6E) resulted in significant Nrf2 protein elevation (Figure 6E), suggesting Nrf2 cascade activation. DEX-induced viability reduction (Figure 6F) and cell death (Figure 6G) were largely inhibited in ko-Keap1 OB-6 cells. Importantly, in ko-Keap1 OB-6 cells adding FGF23 failed to further increase Nrf2 protein accumulation (Figure 6E), nor it can offer additional osteoblast cytoprotection against DEX (Figure 6F and 6G).

These studies further support that Nrf2 cascade activation is required for FGF23-inducd osteoblast cytoprotection.

## DISCUSSION

Xiao et al., have shown that FGF23 could activate FGFR1-Akt signaling without full length α-Klotho [41]. The results of this study suggest that the functional FGFR1 is expressed in OB-6 osteoblastic cells and primary murine osteoblasts. FGF23 treatment *in vitro* induced phosphorylation of FGFR1 and its downstream Akt-S6K1 in OB-6 cells and murine osteoblasts. FGFR1 silencing in OB-6 cells, by targeted shRNAs, largely inhibited FGF23-induced Akt-S6K1 phosphorylation, but augmented with ectopic overexpression of FGFR1. Importantly, FGF23-induced osteoblast cytoprotection against DEX is mediated by FGFR1-Akt signaling. With FGFR1 silencing (by shRNA), Akt inhibition (LY294002) or Akt1 KO (by CRISPR/Cas9d) FGF23 was ineffective against DEX-induced cell death and apoptosis. Conversely, forced Akt activation, by caAkt1, mimicked FGF23’s action and inhibited DEX-induced cytotoxicity in osteoblasts. These results suggest that FGF23 activated FGFR1-Akt signaling to protect osteoblasts from DEX-induced cell death and apoptosis.

DEX treatment in cultured osteoblastic cells/osteoblasts shall provoke significant ROS production and oxidative injury, which is responsible for subsequent cell death and apoptosis. Contrarily, ROS scavengers or oxidative stress inhibition could protect osteoblastic cells/osteoblasts from DEX-induced cell death [39, 42, 43]. Our group and others have indicated that forced activation of Nrf2 cascade, using genetic or pharmacological strategies, can efficiently protect osteoblastic cells/osteoblasts from DEX [9, 10, 37–39, 44]. Li et al., found that a novel Akt activator SC79 activated Akt-dependent Nrf2 signaling to protect osteoblastic cells from DEX [39]. Through activating Nrf2 signaling, an AMP-activated protein kinase activator compound 991 protected MC3T3-E1 osteoblastic cells and primary murine osteoblasts from DEX-induced cell death [37]. Similarly, Icariside II activated Akt-Nrf2 signaling cascade, thus protecting osteoblasts from DEX [10]. Nrf2 activation, via genetic strategies, also offered osteoblast cytoprotection against DEX [9, 38, 44].

However the uses of traditional Nrf2 activators are limited due to their high-concentrations and possible off-target toxicities. In the present study we showed that FGF23, at only ng/mL concentrations, induced significant Nrf2 cascade activation, causing Nrf2 protein stabilization, expression of Nrf2-pathway genes and an increase of NQO1 activity in OB-6 cells and primary murine osteoblasts. Functional studies showed that FGF23 efficiently attenuated DEX-induced oxidative injury in osteoblasts, suppressing superoxide accumulation, lipid peroxidation and mitochondrial depolarization. Therefore, Nrf2 signaling activation by FGF23 exerted anti-DEX osteoblast cytoprotection in osteoblasts.

Studies have implied that Akt (and its downstream mTOR) could be an important upstream molecule of Nrf2 signaling cascade. Lee et al., found that Nrf2 activation by sulforaphane was dependent on activation of the upstream PI3K-Akt [45]. Xu et al., have shown that PI3K-Akt activation is required for pyocyanin-induced Nrf2 activation [46]. Zhang et al., demonstrated that salvianolic acid A (Sal A)-activated Akt phosphorylated Nrf2 at Ser-40 in retinal pigmentation epithelial (RPE) cells, causing Nrf2 protein stabilization and activation [47]. Here we discovered that Akt activation mediated FGF23-induced Nrf2 signaling activation in osteoblasts. LY294002, the Akt inhibitor, or Akt1 knockout abolished FGF23-induced expression of *HO1* and *NQO1* in OB-6 cells. Contrarily, *HO1* and *NQO1* expression was significantly increased in OB-6 cells with caAkt1. These results indicated that FGF23-induced Akt activation severed as the upstream signaling for Nrf2 cascade activation in OB-6 cells.

## CONCLUSION

Collectively, these results suggest that FGF23 activates FGFR1-Akt signaling cascade to protect osteoblasts from DEX-induced oxidative injury and cell death.

## MATERIALS AND METHODS

### Chemicals and reagents

FGF23, DEX, LY294002, Trizol reagents, polybrene and puromycin were obtained from Sigma-Aldrich Chemicals Co. (St Louis, MO, USA). The cell culture reagents were provided by Gibco Co. (Shanghai, China). FGFR1 antibody (#3472) and all other antibodies were purchased from Cell Signaling Technology (Danvers, MA, USA).

### Cell culture

OB-6 human osteoblastic cells [33] and primary murine osteoblasts were differentiated and cultured as described previously [33, 48]. The protocols of this study were approved by IACUC and Ethics committee of Nanjing Medical University. Osteoblast cell differentiation was induced by changing to media containing 10% FBS supplemented with BMP-4 (100 ng/mL).

### Western blotting

After treatment of cells, total cellular lysates (30-40 μg per lane) were separated by 10% SDS-PAGE gels, thereafter transferred onto polyvinylidene difluoride (PVDF) blots. Afterwards, the blots were blocked and subsequently incubated with the applied primary and secondary antibodies. Enhanced chemiluminescence (ECL) reagents (Pierce, Shanghai, China) were utilized to visualize the targeted bands (based on the molecular weights) using x-ray films [13–15]. An ImageJ software (from NIH) was utilized for data quantification.

### FGFR1 shRNA

From Shanghai Genechem Co. (Shanghai, China) a set of non-overlapping lentiviral shRNAs (“Seq1/Seq2”) against human FGFR1 (Targeted sequences, Seq1: 5′- AGTGGCTTATTAATTCCGATA-3′, and Seq2: 5′-GCTTGCCAATGGCGGACTCAA-3′.) were designed and synthesized. The shRNA lentivirus was added to cultured OB-6 cells for 48h. Puromycin (5 μg/mL, in complete medium) was then added to select stable cells for another 6 days. FGFR1 silencing was confirmed by Western blotting. Control OB-6 cells were infected with lentiviral scramble control shRNA (“sh-C”, Santa Cruz Biotech, targeted sequence 5′-GCAAGCTGACCCTGAAGTTCAT-3′).

### Forced FGFR1 overexpression

The full-length FGFR1 cDNA was synthesized by Shanghai Genechem Co. It was inserted into a GV369 construct (Shanghai Genechem Co., Ciha). The construct and the lentivirus-packing plasmids (psPAX2 and pMD2.G, Shanghai Genechem Co., China) were co-transfected to HEK-293T cells, establishing FGFR1-expressing lentivirus (“lv-FGFR1”). The viruses were enriched, filtered, and added to cultured OB-6 cells (cultured in complete medium with polybrene) for 24h. Puromycin was added in the complete medium for 6 days to select stable cells. FGFR1 over-expression in the resulting stable cells was verified by Western blotting.

### CCK-8 viability

Cells were initially seeded into the 96-well plates at 4, 000 cells per well. After the applied DEX treatment, a Cell Counting Kit-8 (CCK-8, Dojindo Laboratories, Kumamoto, Japan) assay kit [49] was utilized to examine the cell viability, with CCK-8 optic density (OD) values tested at 450 nm.

### Lactate dehydrogenase (LDH) assay

As described previously [13–15], following the applied DEX treatment cell death was tested by the LDH assay, using a commercial available two-step LDH assay kit (Takara, Tokyo, Japan). The medium LDH contents were always normalized to the total LDH contents.

### TUNEL (terminal deoxynucleotidyl transferase dUTP nick end labeling) staining

OB-6 cells or the primary murine osteoblasts were initially seeded into the 24-well tissue-culture plates (at 1.2 x 10^4^ cells per well). Following the applied DEX treatment, a TUNEL In Situ Cell Death Detection Kit (Roche Diagnostics Co., Shanghai, China) was applied to quantitatively test cell apoptosis [50]. Cells were co-stained with TUNEL and DAPI, visualized under a confocal fluorescent microscope (Leica, Shanghai, China). For each treatment at least 600 cells in six random views (1×200 magnification) were included to calculate TUNEL ratio (% *vs*. DAPI).

### Mitochondrial depolarization

In cells with mitochondrial depolarization the fluorescence dye JC-1 shall aggregate in the mitochondria, forming green monomers [51]. OB-6 cells or the primary murine osteoblasts were initially seeded into the 24-well tissue-culture plates. Following the applied DEX treatment, cells were stained with JC-1 (3.5 μg/mL, Sigma), washed and tested under a fluorescence spectrofluorometer (F-7000, Hitachi, Japan) at test-wavelength of 545 nm (green).

### Quantitative real-time PCR (qPCR)

The total cellular RNA (extracted through the Trizol reagents) were reverse-transcribed (RT). qPCR was performed by the SYBR green kit under the ABI-7600 fast-PCR system (Applied Biosystems). The 2^ΔΔCt^ method was utilized to quantify expression of targeted mRNAs, using GAPDH as the internal control. All the primers for Nrf2 pathway genes were provided by Dr. Jiang at Nanjing Medical University [52–54].

### Superoxide detection

OB-6 cells or the primary murine osteoblasts were initially seeded into the 96-well tissue-culturing plates (4 × 10^3^ cells per well). Following the applied DEX stimulation, a superoxide colorimetric assay kit (BioVision, Shanghai, China) was utilized to examine the cellular superoxide contents using the attached protocols, with the superoxide’s absorbance tested at the 450 nm [52].

**Lipid peroxidation**

As reported previously [52] OB-6 cells or the primary murine osteoblasts were seeded into six-well plates (1 × 10^5^ cells per well). Following the applied DEX stimulation, the lipid peroxidation assay kit (Abcam, Shanghai, China) was utilized to examine and quantify cellular lipid peroxidation levels, tested by the thiobarbituric acid reactive (TBAR) concentration using the described protocols [52, 55].

### NQO1 activity

Testing the relative NQO1 activity in osteoblastic cells or murine osteoblasts with or without DEX treatment was described previously, using menadione as the substrate [56]. NQO1 activity was always normalized to that of untreated control cells.

### Constitutively-active mutant Akt1

The recombinant adenovirus constitutively-active Akt1 (caAkt1, S473D) construct was provided by Dr. Zhang [36]. Ad-caAkt1 virus or the empty vector (Ad-GFP) virus was added to the cultured OB-6 cells. GFP sorting was performed to select stable cells. caAkt1 expression in the selected single stable cells was verified by Western blotting.

### Akt1 knockout

The lenti-CRISPR-GFP Akt1-KO construct was provided by Dr. Zhang at Soochow University [35]. OB-6 cells were cultured in six well plates, transfected with CRISPR/Cas9 Akt1-knockout construct. Stable cells were selected *via* GFP FACS sorting. Akt1 knockout (KO) in the single stable cells was verified by Western blotting.

### Nrf2 shRNA

The Nrf2 shRNA lentiviral particles (sc-37030-V, from Dr. Xu [40]) were added to OB-6 cells for 24h. Puromycin (5.0 μg/mL) was added to select the stable cells (for 6 days), with 95% Nrf2 knockdown efficiency reached.

### Nrf2 knockout

The lenti-CRISPR-GFP-Nrf2 knockout (KO) construct (from Dr. Xu [40]) was transfected to OB-6 cells via the Lipofectamine 2000 reagents (Thermo-Fisher Invitrogen, Shanghai, China). FACS-mediated selection of GFP-positive OB-6 cells were performed, and the monoclonal stable cells cultured for eight consecutive days. Nrf2 KO was verified by qPCR and Western blotting assays.

### Keap1 knockout

The lentivirus with Keap1 CRISPR/Cas9 KO plasmid was provided by Dr. Liu at Jiangsu University [57], added to cultured OB-6 cells in polybrene medium. After 48h, cells were cultured in puromycin (2.0 μg/mL)-containing medium to establish the monoclonal stable cells, where Keap1 KO was verified by Western blotting.

### Statistical analysis

Experiments were repeated at least three times throughout the study. Quantitative data were expressed as mean ± standard deviation (SD). Statistics were analyzed by two-way ANOVA using a Scheffe’s f-test (SPSS 21.0). To test significance between two treatment groups, a two-tailed unpaired T test (Excel 2007) was utilized. p values < 0.05 were considered statistically significant.

## Supplementary Material

Supplementary Figure 1
