# Supplementary material for: FGF23 protects osteoblasts from dexamethasone-induced oxidative injury
Source: Aging (Albany NY). 2020 Oct 14;12(19):19045–59. doi: 10.18632/aging.103689 (PMC7732311; doi:10.18632/aging.103689)
Supplement: Supplementary Figure 1 [file aging-12-103689-s001..pdf]

SUPPLEMENTARY FIGURE

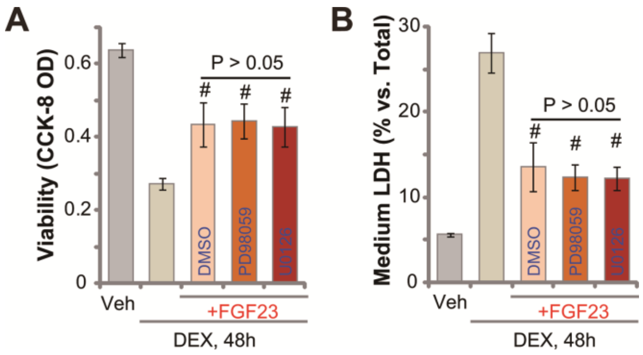

Supplementary Figure 1. OB-6 cells, pre-treated with PD98059 or U0126 (each at 5 μM, 30 min pretreatment), were treated with FGF23 (25 ng/mL) for 1h, followed by DEX (1 μM) or the vehicle control ("Veh") treatment for 48h, cell viability (**A**) and cell death (**B**) were tested. Data were mean ± standard deviation (SD, n=5). $^{\#}$ p<0.05 vs. DEX only treatment. Each experiment was repeated three times and similar results were obtained.
